# Iron physiological requirements in Chinese adults assessed by the stable isotope labeling technique

**DOI:** 10.1186/s12986-018-0262-2

**Published:** 2018-04-20

**Authors:** Jie Cai, Tongxiang Ren, Yuhui Zhang, Zhilin Wang, Lingyan Gou, Zhengwu Huang, Jun Wang, Jianhua Piao, Xiaoguang Yang, Lichen Yang

**Affiliations:** 1 The Key Laboratory of Trace Element Nutrition of The Ministry of Health, National Institute for Nutrition and Health, Chinese Center for Disease Control and Prevention, 29 Nan Wei Road, Xicheng District, Beijing, 100050 China; 2 National Institute of Metrology, National Research Center for Certified Reference Material, No.18, Bei San Huan Dong Lu, Chaoyang Dist, Beijing, People’s Republic of China; 3 Bethune Military Medical College, Zhongshanxi Road 450, Shijiazhuang, Hebei province China

**Keywords:** Iron physiological requirements, Chinese adults, Stable isotope labeling technique

## Abstract

**Background:**

Iron is a kind of essential trace mineral in the human body, while the studies on its physiological requirement are very limited recently, especially in China. And most studies were performed with the radioisotope tracer technique, which was harmful to health. This study aimed to first get the value of iron physiological requirements in Chinese adults assessed by the stable isotope labeling technique.

**Methods:**

Forty-four eligible young Chinese healthy adults were randomly recruited from the Bethune Military Medical College (Shijiazhuang, Hebei, China) between January 2010 and March 2011, and 19 subjects were included in the final data analysis. After adaptive diets and observation, subjects received ^58^Fe intravenously. The baseline venous blood sample and general information were collected on day 0. Venous blood samples were also collected on day 14, 30, 60, 100, 120, 150, 240, 330, 425, 515, 605, 767, 1155, respectively. The blood samples were acid digested by a Microwave Digestion System and then analyzed by the MC-ICP-MS and Atomic Absorption Spectroscopy to get the abundance of Fe isotopes and the total iron concentration respectively. The circulation rate (the proportion of blood iron to whole body iron) could be calculated by the intake amount, background content and the peak isotope content. When the abundance changed stably, the iron physiological requirement could be calculated by the iron loss in a period of time.

**Results:**

The abundance of ^58^Fe reached its peak on day 14, and changed stably from day 425. The average circulation rate was 84%, with no significance difference between the 2 genders. The mean iron requirement in females was 1101.68 μg/d, and the mean requirement adjusted by body weight was 20.69 μg/kg.d. For males, the mean iron requirement was 959.9 μg/d, and the requirement adjusted by body weight was 14.04 μg/kg.d.

**Conclusion:**

Our study has obtained the data about the iron physiological requirements of Chinese adults using stable isotope labeling technique, which could provide the basis for adjusting iron DRIs of Chinese people in the future.

**Trial registration:**

The trial was registered at the Chinese Clinical Trial Registry (No: ChiCTR-TRC-09000581).

## Background

Iron is the most abundant trace mineral in the human body, with an average of 3–4 g present in adults [1]. The main form of dietary iron intake in most countries is non-heme iron, which has a low absorption and utilization rate. Iron deficiency anemia (IDA) can lead to developmental delays, behavioral disturbances, perinatal complications, and the impairment of learning ability and cognitive function [2, 3], which affects approximately one-quarter of the world’s population [4] and 9.7% of the population in China [5]. Meanwhile, iron overload may lead to various pathological clinical outcomes, such as pancreatic damage, cardiovascular disease, neurological disease and cancer [6–8].

Almost two-thirds of body iron is found in the hemoglobin present in circulating erythrocytes, other is contained in muscle tissue, iron store, and in variety of enzymes involved in oxidative metabolism and many other cell functions [9]. Evidence shows that iron balance in humans is dependent on variations in absorption other than excretion from the body [10, 11]. The physiological requirement in adult males who have no significant change in weight is usually regarded equal to iron loss [12]. The iron requirement value is the core parameter of dietary reference intakes (DRIs), which could help people to reasonably plan their iron intake and maintain iron homeostasis.

The traditional methods used to research iron loss in adults are those such as the factorial method of calculating nutrient requirements, metabolic balance and radioisotope labeling method. The factorial calculation is an indirect method based on calculation which adds up all aspects of iron requirements. Metabolic balance is a classical method in the field of nutrition which requires simple testing equipment and easy operation. The balance test is often unable to obtain true and reliable data because the iron absorption rate may be either overestimated or underestimated [13]. The radioisotope tracer technique is simple, accurate, highly sensitive, and can be adopted during the normal physiological conditions of an organism. Since 1939, a series of studies on the iron requirements in human adults using the radioisotope tracer method have been carried out in western countries [14]. However, the radiation effect and finite choice of radioactive isotopes have limited their more application. With the development of technology, the stable isotope labeling technique, with its advantage of no radioactive health hazards to subjects, has been used for studying absorption and utilization rate of iron in recent years [15, 16], while rarely used in iron physiological requirements. A study using ^58^Fe by Fomon in 2005 only focused on toddlers [17]. In studies conducted using radioactive isotopes, most of the subjects were adult males [18, 19], and the figures about women were very limited. In China, there is no direct experimental data from the domestic population and the values related to DRIs are all calculated based on experimental data from other countries.

In this study, we aimed to firstly obtain the value of iron physiological requirements in Chinese adults. Evidence shows that most iron in the body is combined with hemoglobin and exists in the circulatory system [10]. Iron physiological requirements could be calculated according to the change of isotope abundance in the blood. This experimental data related to the iron nutrition of Chinese adults can reflect the iron requirement of the Chinese population more accurately and help us to better update and adjust the DRIs in China. And this method for evaluating iron physiological requirement can be referred in the further studies.

## Methods

### Location and experimental design

We assumed that the proportion of blood iron to whole body iron, the circulating rate (usually called the erythrocyte incorporation rate in many studies), was approximately constant during the observation period. Then, the change of iron isotopic abundance in blood is consistent with the change in whole body. After the subjects had taken a certain amount of enriched ^58^Fe, the isotope content in the blood circulation reached its peak in a relatively short time. At this time, the circulating rate of iron was calculated according to the amount of the isotope absorbed and the content of the isotope in blood. Thereafter, the ^58^Fe abundance in the blood gradually became diluted by the natural isotopic composition of iron having a higher abundance of ^56^Fe than the blood. The smooth transition of abundance is known as the “stable period”, marking the point when the labeled ^58^Fe is fully mixed into the body. At this point, changes of iron isotopic abundance in blood can represent changes in the whole body. So, monitoring for over 3 years ensured that isotopic abundance had reached the stable period. Iron loss in circulation for a specific period of time during the stable period was obtained by accurately measuring the change ^58^Fe abundances in the blood. In this way, the total iron loss in the human body, which is physiological requirement, was calculated combined with the circulating rate.

Forty-four eligible young Chinese healthy adults (22 women and 22 men) were randomly recruited from the Bethune Military Medical College (Shijiazhuang, Hebei, China) between January 2010 and March 2011. Subjects had not taken any mineral or vitamin supplements within 1 month before the study. Subjects were excluded under the following conditions: 1. those who were infected with a disease that could affect iron absorption and metabolism (such as malabsorption, ulcer and inflammatory diseases) or under abnormal iron nutrition statuses; 2. those who regularly took medication that could affect iron absorption and metabolism; 3. women during pregnancy or menstruation.

At the beginning of the trial, the subjects received isotope ^57^Fe and ^58^Fe (the majority of ^57^Fe orally and ^58^Fe intravenously). By monitoring the first 14 days, our team obtained the absorption rate and the red cell utilization rate of non-heme iron, and the results of male have been published. Specific diet recipes and the preparation of isotopically labeled iron have also been described in detail in the previous article [20]. The women and men underwent the same procedure in this study, while the current article on women has not yet been published. Now, this article focused on the physiological requirements of iron, which was got by more than 3 years of monitoring the isotope abundance in subjects diluted. Baseline venous blood samples and general information were collected on day 0. Venous blood samples were also collected on day 14, 30, 60, 100, 120, 150, 240, 330, 425, 515, 605, 767 and 1155, respectively. Throughout the follow-up, attention was paid to the health condition of all subjects to ensure that there were no traumatic blood loss and abnormal iron metabolism. The female menstrual period was avoided in the first 14 days of the experiment, while it could not be avoided for the whole follow-up over the 3 years. After the preliminary experiment, ^58^Fe was considered more appropriate for the research of iron physiological requirements for its lower natural abundance and less interference. So we used only the abundances of ^58^Fe in this part of study. The trial was registered at the Chinese Clinical Trial Registry (No: ChiCTR-TRC-09000581) and approved by the Ethics Committee of the National Institute of Nutrition and Health, Chinese Center for Disease Control and Prevention. The informed consent was also obtained from all subjects prior to participation.

### Analytical method

Venous blood samples were collected by evacuated tubes containing K2-EDTA (BD, New Jersey, USA) and stored in a refrigerator (− 80°C). Before measuring, the blood samples were acid digested by a Microwave Digestion System (Mars 6, GEM, USA). The 0.5 g blood sample was mixed with 6 ml 70% HNO_3_ solution following the digestion procedure (120°C: ramp 6 m, hold 5 m; 150°C: ramp 5 m, hold 15 m; 190°C: ramp 5 m, hold 30 m; 1600 W). The abundance of Fe isotopes was analyzed by the Multi-collector inductively-coupled plasma mass spectrometry (MC-ICP-MS) with a standard-sample bracketing method. All Fe isotopes were simultaneously detected together with ^60^Ni as a monitor for the correction of isobaric interferences of ^58^Ni on ^58^Fe in one sequence. A mixture of argon and H_2_ were used as collision gas to eliminate the interferences [21]. Under optimized conditions, external precision of the order of 0.01–0.03% (relative standard deviation, RSD), and 0.1–0.3% (RSD) was obtained for ^54^Fe/^56^Fe, ^57^Fe/^56^Fe and ^58^Fe/^56^Fe, respectively. The abundance of ^58^Fe were measured accordingly. Total iron concentration analysis was performed by the Atomic Absorption Spectroscopy (AAS) (PinAAcle 900, PerkinElmer). Biochemical assays for iron status of the serum ferritin (SF), unsaturated iron-binding capacity (UIBC), serum iron (SI), serum transferrin receptor (sTfR) and inflammation markers of C-reactive protein (CRP) were performed using an automatic biochemical analyzer (Hitach7180, Japan).

### Calculation method

After the iron isotopes ^58^Fe taken in test days were completely mixed with the iron in the body of the subjects and reached a stable state, the daily loss of iron was calculated by the change in iron isotope during a period of time (assuming day i to day i + t). Since the subjects were in iron homeostasis, the total amount of iron in the body remained approximately constant, which meant the amount of daily requirement was relative invariant. According to the report by the USA National Academy Press in 2001, for the healthy men in iron homeostasis, the daily loss was equal to the daily requirement (assuming R in the formula) [12]. For women, blood loss in the menstruation would fluctuate the iron status in several days. To balance this effect, we collected blood samples away from their menstrual periods and chose a relative long period of time for calculation.

So, the isotopic content of iron in the whole body on day i minus the loss in t days, plus the isotope of iron absorbed with natural abundance in t days, was equal to the isotopic content of iron in the whole body on day i + t. The formula can be deduced as follows:

1-a $$ \mathrm{T}\times \mathrm{V}\times {\mathrm{P}}_{\mathrm{i}}\div \mathrm{C}-\mathrm{t}\times \mathrm{R}\times \left({\mathrm{P}}_{\mathrm{i}}+{\mathrm{P}}_{\mathrm{i}+\mathrm{t}}\right)/2+\mathrm{t}\times \mathrm{R}\times \mathrm{NA}=\mathrm{T}\times \mathrm{V}\times {\mathrm{P}}_{\mathrm{i}+\mathrm{t}}\div \mathrm{C} $$

After formal transformation:

1-b $$ \mathrm{R}=\mathrm{T}\times \mathrm{V}\times \left({\mathrm{P}}_{\mathrm{i}}-{\mathrm{P}}_{\mathrm{i}+\mathrm{t}}\right)\div \mathrm{t}\div \left[\left({\mathrm{P}}_{\mathrm{i}}+{\mathrm{P}}_{\mathrm{i}+\mathrm{t}}\right)/2-\mathrm{NA}\right]\div \mathrm{C} $$

T: the concentration of total iron in the blood (mg/L).

P_i_: the isotopic abundance on day i.

NA: the natural abundance of isotopes.

t: total number of days in the period of time which recorded for calculation.

R: daily loss or intake of iron (mg).

C: the circulation rate (the proportion of iron in the blood to total iron in the body).

V: blood volume (L), which was by the formula published by Carlsen and Bruun [22], V (ml) = (45.2 + 25.3 × exp.(− 0.0198 × DDW)) × BW (kg), where DDW = 100 × (BW(kg) − 7.582 × exp.(0.01309 × BH (cm))) / 7.582(0.01309 × BH (cm)).

The circulation rate can be calculated by the quantity of administered isotope in the blood and the total amount of administered isotope. The administrated ^58^Fe in the blood was calculated by the following formula described in the article by Steven A Abrams [23]:

2 $$ {}^{58}{\mathrm{Fe}}_{\mathrm{inc}}=\left[{}^{58}\mathrm{Fe}/{}^{56}{\mathrm{Fe}}_{\mathrm{enr}}-{}^{58}\mathrm{Fe}/{}^{56}{\mathrm{Fe}}_{\mathrm{base}}\right]\times {\mathrm{Fe}}_{\mathrm{circ}}\times {\mathrm{NA}}_{58}\div \left({}^{58}\mathrm{Fe}/{}^{56}{\mathrm{Fe}}_{\mathrm{base}}\right) $$

^58^Fe_inc_: the quantity of administered ^58^Fe in the blood (mg).

^58^Fe/^56^Fe_enr_: ^58^Fe/^56^Fe on its peak day (day 14) measured by MC-ICP-MS.

^58^Fe/^56^Fe_base_: ^58^Fe/^56^Fe on day 0 measured by MC-ICP-MS.

Fe_circ_: the total iron in circulation (mg), which was calculated by blood volume and concentration of total iron in the blood measured by AAS.

NA_58_: the natural abundance of ^58^Fe.

### Statistical analysis

General statistical analysis was performed using SPSS version 19.0. The normality of the data distribution of the tests under study was investigated with the Kolmogorov-Smirnoff test. CRP and sTfR did not obey normal distribution, so they were expressed as median (P25, P75), other variables were expressed as means ± standard deviations ($\overline{\mathrm{X}}\pm SD$). The differences between the two genders were compared by t test. The time when the change of isotopic abundance became stable was determined by the Repeated Measures Analysis of Variance Analysis (RMANOVA) combined with the line chart of the change. Comparison of iron nutritional status at different time points was also performed by RMANOVA. The rank sum test was used when comparing CRP and sTfR on different days. The t test and RMANOVA were performed by SAS version 9.4, and *P* values less than 0.05 were considered statistically significant.

## Results

### Identification of the stable period

After receiving ^58^Fe, follow-up monitoring of the subjects was conducted for more than 3 years. Twenty subjects (10 female and 10 male) with complete data of 13 blood samples (day 0, 14, 30, 60, 100, 120, 150, 240, 330, 425, 515, 605, 767) were used to identify the stable period, and their information is presented in Table 1. The mean age of the subjects was 22, ranging from 20 to 25. The mean height, weight and BMI was 167.55 cm, 57.7 kg and 20.48 kg/cm^2^, respectively.

**Table 1:** The demographic information of subjects with complete data

	n	age	height(cm)	weight(kg)	BMI(kg/cm^2^)	^58^Fe received(mg)
female	10	22 ± 1	163.40 ± 3.37	51.3 ± 4.9	19.20 ± 1.80	3.70 ± 0.17
male	10	23 ± 1	171.70 ± 3.86	64.1 ± 8.9	21.75 ± 2.93	4.93 ± 0.52
total	20	22 ± 1	167.55 ± 5.53	57.7 ± 9.6	20.48 ± 2.70	4.31 ± 0.73

*BMI* Body Mass Index

**Fig. 1:**
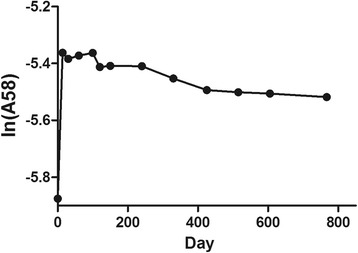
The abundance change of ^58^Fe

On day 0, the mean abundance of ^58^Fe was 0.002809, which was very close to the natural abundance. Because the decrease of isotopic tracer in the blood after the stable period is exponential, the abundance of ^58^Fe was log-transformed (natural logarithm) to help judgment. The changes of isotopic abundance (ln A(^58^Fe)) in blood are shown in Fig. 1. After the injection of artificially enriched ^58^Fe, its abundance in the blood peaked dramatically (on day 14). The curve then fluctuated for a period of time until eventually stabilizing. From observation, we found the change of ^58^Fe became smooth and reached the stable period from day 425.

Polynomial comparison of RMANOVA was also used to help determine the stable period (Table 2). Some periods of time with the log-transformed abundance were intercepted to see if the first and second degree polynomial contrast were meaningful in the comparison. The results showed that the curve appears a linear trend from day 120-767, while the curve trend also came from day 240-767. Until day 330, the real linear trend appears. And from day 425, the abundance changed so slightly that the linear trend was not recognized by SAS. Therefore, it was conservative to consider that the stable period started from day 425 through synthetical consideration.

**Table 2:** *P* values of polynomial contrast by RMANOVA

	start(day)	60	100	120	150	240	330	425	515
	over (day)	767	767	767	767	767	767	767	767
Ln(A^58^Fe)	time_1*	< 0.0001	< 0.0001	0.0009	0.0014	0.0004	0.0004	0.2178	0.4797
	time_2*	0.0321	0.0063	0.0748	0.0699	0.0457	0.2600	0.9708	0.9450

*time_N represents the nth degree polynomial contrast for time

### The circulation rate

The abundance of ^58^Fe in the blood peaked on day 14. The circulation rates of subjects are shown in Table 3. In this part, of the 44 subjects enrolled, 1 subject dropped out before day 14, while the results of 3 subjects were significantly abnormal and removed of the final statistical analysis. The results of 40 subjects were included. The average circulation rate was 84.06%, with no significance difference between the 2 genders.

**Table 3:** The circulation rate of subjects

	n	weight (kg)	age	circulation rate(%)
female	21	54.6 ± 6.82	22.24 ± 1.03	82.80 ± 12.55
male	19	67.43 ± 12.20	22.56 ± 1.00	85.46 ± 7.71
total	40	60.7 ± 11.60	22.38 ± 1.02	84.06 ± 10.49

### The iron physiological requirements

**Fig. 2:**
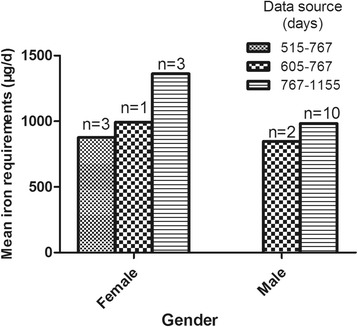
The iron requirements

**Fig. 3:**
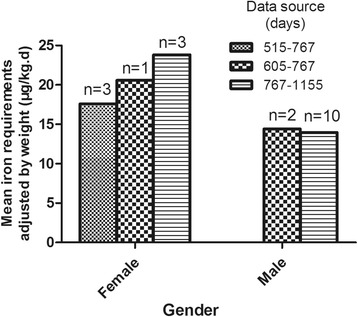
The iron requirements adjusted by weight

Because the stable period started from day 425, we used the data after day 425 to calculate the iron daily requirement. Until day 425, 18 subjects could not be monitored for follow up (due to leaving the study, loss of contact, inability to obtain a blood sample, etc), and the abundance curves of 5 subjects changed abnormally (due to unknown reasons, excluding trauma, bleeding, and abnormal metabolic conditions) and the data from them could not be included for calculation. Twenty-one subjects was included for calculation, and 2 outliers were eliminated (out of mean ± 3 standard deviation). Ultimately, the results of 19 subjects were included in the final data analysis. The iron daily requirement of subjects was calculated by the formula (1-b), with the data on day 515, 605, 767 and 1155. Due to the difficulty in sample collection and cohort following for such a long time, 4 lots of blood samples were not received from every subject. In theory, the average daily loss of iron should be approximately equal at different periods of time after the stable period was reached. Thus, we selected the most suitable period of time for calculation according to the abundance curve of every subject. The calculation results from different data sources are shown in Table 4, and mean requirements between 2 genders are also compared in Table 5. Bar charts describing the iron requirements from different gender and data sources are illustrated in Figs. 2 and 3.

**Table 4:** The iron physiological requirements of different data sources

gender	data source^a^	n	mean body weight (kg)	iron requirements (μg/d)			iron requirements adjusted by weight (μg/kg.d)		
				mean ± std	comparison of different data source		mean ± std	comparison of different data source	
					F	P		F	P
female	A	3	49.98 ± 3.59	877.11 ± 352.09	2.37	0.209	17.59 ± 7.13	0.594	0.594
	B	1	48.28	993.4			20.58		
	C	3	59.28 ± 12.31	1362.34 ± 175.52			23.83 ± 6.9		
male	B	2	58.76 ± 0.14	846.65 ± 125.97	0.199	0.665	14.41 ± 2.11	0.018	0.896
	C	10	69.1 ± 13.08	982.55 ± 412.39			13.97 ± 4.38		

^a^A: day 515 to day 767, B: day 605 to day 767, C: day 767 to day 1155

**Table 5:** The iron physiological requirements between 2 genders

gender	n	mean body weight (kg)	iron requirements (**μ**g/d)			iron requirements adjusted by weight (**μ**g/kg.d)		
			mean ± std	comparison of different genders		mean ± std	comparison of different genders	
				T	P		T	P
female	7	53.72 ± 9.06	1101.68 ± 335.76	0.819	0.424	20.69 ± 6.53	2.771	0.013
male	12	67.38 ± 12.5	959.9 ± 378.66			14.04 ± 4.02

The mean body weight was 53.72 kg for females and 67.38 kg for males, with significant difference between the 2 genders (*t* = − 2.517, *P* = 0.022). The mean iron requirement in females was 1101.68 μg/d, and the mean requirement adjusted for body weight was 20.69 μg/kg.d. Both results from different data sources showed no significant difference. For males, the mean iron requirement was 959.9 μg/d, and the requirement adjusted for body weight was 14.04 μg/kg.d. Again, there was no significant difference from different data sources. The mean iron daily requirement adjusted for body weight showed significant difference between the two genders (*t* = 2.771, *P* = 0.013).

Biochemical assays of iron status (sTfR, SF, UIBC, SI) and inflammation markers (CRP) were measured with the serum on day 0, 767 and 1155. Throughout the study, the iron status of subjects were about normal. And for each index, there is no statistical difference of different time points (*P* > 0.05) (Table 6).

**Table 6:** Iron nutritional status

Day	n	SF(ug/L)	UIBC(umol/L)	SI(umol/L)	sTfR(g/L)	CRP(mg/L)
0	19	74.8 ± 54.5	24.7 ± 9.4	17.8 ± 6.1	2.6 (2.19,3.08)	0.1 (0,0.3)
767	17	101.3 ± 54.5	27.2 ± 8.2	18.5 ± 5.8	2.21 (1.94,2.35)	0.3 (0.1,0.5)
1155	12	103.4 ± 44.2	29 ± 7.8	20.9 ± 6	2.37 (2.17,2.6)	0.5 (0.2,0.8)
Statistics		F = 2.787	F = 0.502	F = 1.971	χ^2^ = 1.000	χ^2^ = 4.471
*P* value		0.092	0.615	0.190	0.607	0.107

## Discussion

To obtain the more reliable result, we assumed that the total amount of body iron of each subject was stable during the whole study. If the object was in the condition of bleeding, pregnancy and childbirth, it might affect the metabolism and amount body iron. In case the misestimates caused by these conditions, we have monitored the iron nutritional status of all subjects and ensured that they were all in the iron homeostasis. In order to ensure that the ^58^Fe was fully mixed in the body, we combine the result of RMANOVA with the curve trend, and finally chose the more conservative result.

The change of Fe isotopic abundance in blood indirectly reflected the distribution and metabolism of iron in the body. The mean abundance of ^58^Fe on day 0 were very close to the natural abundance, proving the accuracy of the detection to a certain extent. After a series of transportation and metabolic processes, the iron intake in the test was fully mixed with the iron in the body and redistributed to a steady state. Some of the iron was bound to transferrin and then transported to the bone marrow for the hemoglobin synthesis in developing erythroid cells, which was the most important iron pool and had the highest turnover. The reticulocytes were released in to circulation from this site and became mature red cells with a lifespan of 120 days [8]. After 120 days, the iron released form the dead red cells would be recycled [10]. Because of these metabolic processes, it took some time for iron to reach the steady state, so we needed to find the stable period and calculate the iron daily requirement afterwards.

Our results show that the stable period started from day 425. A study by Hiroshi Saito et al. in 1964 reached the conclusion that the stable period was from day 360 [19]. In their study, 12 men were enrolled and injected with radioactive isotope ^59^Fe. And in a study used ^58^Fe by Fomon in 2005, the subjects of which were toddlers, the stable period was from the 13th month (nearly day 390) [17]. Our result of day 425 was a little later than their results, which may be due to the different subjects used and experimental design.

The majority of total body iron is present in hemoglobin in circulating erythrocytes [8], while its accurate proportion still lacks consensus. This proportion, which we called the circulating rate, was actually equal to the erythrocyte incorporation rate mentioned in other studies because the formulas were to calculate the ratio of ^58^Fe in the blood compared to the ^58^Fe administrated. In the article published by our group in 2016 [20], the ^58^Fe incorporation rate of males was 85%, consistent to our rate of 85.46%. While in this paper, the concentration of total iron in blood was measured by AAS, which might be more accurate than previous studies using hemoglobin concentration as an estimate. Furthermore, in this paper we used isotopic abundance in whole blood for calculation whilst using isotopic abundance in red blood cell samples in the previous article. Many other studies also obtained the circulating rate. Hiroshi Saito et al. in 1964 enrolled 12 men in a study and reported that the average utilization of radio iron was 90%, and 10% remained in tissues [19]. Larsen L in 1975 reported that mean red cell utilization of absorbed ^59^Fe was 92.9% in adults [24]. Both of these studies used the radioactive isotope ^59^Fe. However, many studies showed that this rate was lower in infants (less than 80%) compared with adults [25]. Zlotkin et al. [26] and McDonald et al. [27] reported that the ^58^Fe incorporation rate was 75% and 68% in preterm infants, respectively. This difference might be explained by the fact that the cell synthesis in infants was more active than that in adults.

Due to the long follow up time and difficulty of continuous blood sample collecting in this study, it was relatively hard to maintain data for all the subjects and the data that could be used for calculation was also limited. Of the 19 subjects finally included in the statistical analysis, the results of 12 males were close to that of Seattle whites (0.95 mg/d, 12.1 μg/kg.d), Durban Indians (1.02 mg/d, 16.4 μg/kg.d) and Araya Mestizos (0.90 mg/d, 13.3 μg/kg.d) in the study by Green et al. in 1968 [18], and the present Chinese DRIs of iron were calculated based on its results, which only reported iron loss in men. Before this study, Finch et al. calculated that daily iron loss was approximately 0.61 mg/d in men (mean weight 66 kg), 0.64 mg/d in non-menstruating women (mean weight 59 kg) and 1.22 mg/d in menstruating women (mean weight 61 kg) [28]. In our data, the real different iron requirements of the 2 genders were covered by the different mean body weight, so a significantly higher requirement for iron appeared in women after adjustments for body weight. In this study, we avoided menstruation during the test days (day 0 to day 14) and pregnancy and fertility during the entire experimental period (day 0 to day 1155), whilst menstruation might not be avoided during the long follow up time. So, the results we calculated from women were supposed to contain the loss during the menstrual period. This might be the main reason for the different requirements between males and females. Our result of women was consistent with that described in the report published by American national academy press in 2001 [12]. In the report, the iron requirements for women were estimated by customary two-component model, consisting of basal losses and menstrual losses. The median iron requirement for women was 1.4 mg/d, while the mean weight of subjects for calculating basal losses was 64 kg. Some studies have reported lower figures than us. Hiroshi Saito et al. in 1964 [19], using a whole body isotopic retention technique, reported that daily iron loss was of 0.89 mg/d in 12 men. In 2005, Fomon et al. used ^58^Fe to research the iron loss of toddlers. From 13 to 26 months of age, the daily inevitable iron loss was 0.25 mg/d (mean weight was 10.3 kg in 13 month and 13.1 kg in 26 month) [17]. This daily iron loss adjusted by weight of toddlers is similar to that of our female subjects. Changes in people’s nutritional status, as well as differences in populations and methods of experimentation, may all contribute to the differences in outcome. Overall, however, our results were approximately in line with other studies. The main limitation of our study was that fewer cases could be used for calculation, especially for women. We will further validate the results in the future.

## Conclusion

Our study firstly used the stable isotope technique to study iron physiological requirements. We obtained data about the daily iron requirement of Chinese adults, relatively consistent with other studies, which could provide the basis for adjusting iron DRIs in the future.

## Abbreviations

AAS: Atomic Absorption Spectroscopy
CRP: C-reactive protein
DRIs: Dietary reference intakes
IDA: Iron deficiency anemia
MC-ICP-MS: Multi-collector inductively-coupled plasma mass spectrometry
RMANOVA: Repeated Measures Analysis of Variance Analysis
RSD: Relative standard deviation
SF: Serum Ferritin
SI: Serum Iron
sTfR: serum Transferrin receptor
UIBC: Unsaturated Iron-Binding Capacity
